# “It doesn’t exist, only other people have it, or it’s bad luck”: perceptions of HIV as barriers to its prevention in Bata

**DOI:** 10.1186/s12889-023-17215-0

**Published:** 2023-11-27

**Authors:** C. Rodríguez-Reinado, T. Blasco-Hernández, N. Abeso, A. Benito-Llanes

**Affiliations:** 1 Department of Sociology, Social Work and Public Health, University of Huelva, Dr. Cantero Cuadrado St., 6. 21004 Huelva, Spain; 2 National Center for Tropical Medicine, Carlos III Health Institute, CIBERINFEC (Infectious Diseases CIBER), Sinesio Delgado St., 8, 28029 Madrid, Spain; 3 Ministry of Health and Social Welfare of the Republic of Equatorial Guinea, Intersectoral Collaboration to End HIV, Malabo, Equatorial Guinea

**Keywords:** HIV, Primary prevention, Perceptions of HIV, Qualitative method, Africa

## Abstract

**Background:**

Currently, Africa is the region of the world where the highest number of new cases of HIV infection are registered. In 2022, Equatorial Guinea was the Central African country with the highest HIV prevalence (6.9%) and incidence (3.80 per 1,000 amongst the population of all ages). The main objective of this study was to determine the perceptions of HIV and the meanings given to it among the population of Equatorial Guinea in order to assess to what extent they represent a barrier to the prevention strategies implemented hitherto.

**Methods:**

A total of 30 semi-structured interviews and nine focal groups were carried out.

**Findings:**

The interviewees’ testimonies revealed a combination of differing perceptions and meanings around HIV. In some cases, HIV was perceived as “a non-existent illness”, and in others as “a disease of others”, or as “a disease of bad luck”. Other majority perceptions of HIV classed it as “a deadly disease” or “a sexual illness”.

**Conclusions:**

All these perceptions of HIV and the social representations constructed around it can represent a barrier to adopting preventive practices. Hence, in order to improve efficacy, efficiency, and effectiveness, it is recommended that HIV prevention policies take into account the heterogeneity of meanings linked to the different social groups that have contracted the virus.

**Supplementary Information:**

The online version contains supplementary material available at 10.1186/s12889-023-17215-0.

## Background

HIV/AIDS is one of the leading causes of mortality and morbidity among the population of Equatorial Guinea [1, 2]. Prevalence studies show a progressive increase of the epidemic in the country in recent decades: 2.3% in 2001, 4.3% in 2012 and 6.4% in 2016 [3–5]. In 2021 figures for HIV/AIDS in the country reached the unprecedented level of 6.9% amongst the population aged 14–49 years [1].

This growing trend can also be seen among pregnant women: 1.5% in 1997, 7.3% in 2008 and 10.2% in 2015 [3, 6]. The incidence rate was 3.80 per 1,000 inhabitants for all ages in 2022 [1]; thus Equatorial Guinea is the Central African country with the greatest comparative increase in incidence since 2010, according to the UNAIDS report for 2022 [1]. For example, it is worth mentioning the case of Burundi, a neighboring country, which achieved an impressive decrease in cases of new HIV infections between 2010 and 2018 [1]. Equatorial Guinea, however, is lagging behind in the results obtained, given the increase of more than 10% in new HIV infections from 2010 to 2018 [7]. These figures take the country even further away from achieving the 2025 goal of reducing new infections by 82.5% since 2010 [8, 9].

The growth of the epidemic in Equatorial Guinea shows that HIV prevention continues to be the key to controlling the epidemic in the country.

### HIV prevention policies in Equatorial Guinea

As in most African countries, the primary prevention strategies implemented in Equatorial Guinea have essentially been based on the development of community health education campaigns around HIV prevention, using an ABC approach (abstinence, be faithful and use condoms), with the aim of preventing new cases of sexual infections [10, 11]. However, the results of a study conducted in this area show that 82.6% of the adolescents interviewed had not heard of sexual abstinence as a method of protection against HIV in Equatorial Guinea [12], and 76.1% were not aware of the male condom [12]. This risk situation is aggravated by the fact that the study also shows that most of the young people interviewed believed that neither fidelity nor sexual abstinence were feasible [12]. Nevertheless, these data contrast with the figures from the UNAIDS report (2022) on the use of the male condom in the last sexual relationship with an unmarried person among people aged 15 to 49 (43.4% in women and 62.6% in men) in the neighboring country of Cameroon [1].

In terms of secondary prevention measures in Equatorial Guinea, the focus has been on mother and child prevention, the extension of voluntary HIV testing to the entire population and the Start Free, Stay Free, AIDS Free 2020 framework [3–5, 13]. In this respect, mother and child prevention seems to have been more successful [14]. While in 2010 the percentage of pregnant women diagnosed with HIV resorting to medication was 16%, it increased to 42% in 2022 [1]. However, when compared to the other Central African countries, Equatorial Guinea and Congo have the worst results [1]. Finally, it is worth mentioning the control of the epidemic through coverage and adherence to antiretroviral treatment. In this regard, one study revealed than 58.20% had low or insufficient adherence to treatment [15]. In addition, there was a significant positive correlation between CEAT-HIV scores and CD4 T-cell levels [15].

### Social and health context of Equatorial Guinea

Equatorial Guinea is one of the countries with the highest per capita income in Africa (GDP: €10,366 million). Its HDI (0.596), however, is below the world average for human development [16]. The maternal mortality rate is 110 deaths per 1,000 live births [17], the infant mortality rate is 57,2 deaths per 1,000 live births in a given year, and the birth health index is 0.57% [18]. Only 56% of households have access to drinking water [19].

In terms of health coverage, despite improvements in government budget allocations to health spending over the last decade [20], much remains to be done. For example, comparing data from the UNAIDS report (2022) on HIV budget allocations, Equatorial Guinea ($7,844,051) still lags behind many Central African countries with lower per capita incomes, such as the Republic of Congo ($18,662,067) [1].

Therefore, in this epidemiological and social context, there is an urgent need to prioritize improvement of the effectiveness of the prevention strategies implemented, with the aim of reversing the curve of the epidemic in Equatorial Guinea. Therefore, this qualitative study is timely and important, as it yields information that will aid the design of prevention strategies and enhance their effectiveness, efficacy and efficiency. The specific objective of the study was to ascertain the meanings and perceptions of HIV among the Fang adult population of Equatorial Guinea, in order to determine whether they represent a barrier to the prevention strategies implemented.

## Methods

The study adopted a qualitative method, using grounded theory [21] (see CORED LIST in Supplementary material). It was conducted from 2014 to 2016 in the capital, Bata, and three neighboring towns (Nkuantoma, Mokomo and Midjimitom). It should be noted that the Guinean population is made up of different ethnic groups [22]. The target population was the Fang ethnic group, since it is the majority ethnic group in the country and continental region (87.5%) [23].

### Sample

The sampling was qualitative. According to the types set up by Teddlie and Yu [24] the sample was stratified by purpose, with the variable of *use or not of HIV diagnosis and treatment services*. From this, two segments of the Fang population were derived: (1) people who had never used HIV diagnosis and treatment services in Bata and therefore did not know if they were HIV-positive or not; and (2) people who had used HIV testing and treatment services in Bata and therefore were HIV positive. Different recruitment channels were utilized: (1) through traditional medicine health professionals; (2) through modern medical professionals; (3) through teachers in the education system. In addition, specific demographic variables were also taken into account: gender, education, age, habitat, health care practices (modern or traditional medicine) and whether or not the person was seropositive for HIV. In terms of sample size, the saturation-sampling criterion was applied. The final sample consisted of 82 participants (Table 1).

**Table 1:** Demographic characteristics of the participants (*n* = 82)

	**Without Diagnosis**	**With Diagnosis**
Gender		
Female	26	15
Male	26	15
Education		
Low (primary and secondary school)	31	15
Medium (tertiary education or vocational training)	12	14
High (diploma and university graduates)	9	1
Habitat		
Urban	26	30
Rural	26	-----
Age		
20–30	4	11
30–40	30	11
40–50	13	4
50–60	5	4
Type of medicine		
Traditional	26	--------
Modern	56	30

### Data collection techniques

Thirty semi-structured interviews lasting 45 to 60 min were conducted among the HIV-positive segment of the population. Using individual semi-structured interviews among the first population segment enabled us to maintain the confidentiality and anonymity of these participants in the context of their illness. Nine focus groups lasting 60 to 90 min were also carried out among the segment of the population that did not know their HIV status.

Both techniques were guided by a thematic script containing study dimensions relevant to the research objectives: (1) perceptions and meanings of health and illness; (2) perceptions and meanings of HIV and AIDS. A review of the scientific literature on the topic was also conducted for the design of the script.

Prior to carrying out the fieldwork, the thematic scripts (Table 2) were piloted through exploratory interviews among people with and without HIV, in order to test the relevance, clarity and comprehensibility of the questions and the appropriateness of the instrument to the cultural context. One expert researcher and one local observer moderated the face-to-face interviews and focus groups. The venue for the interviews varied according to the person interviewed (health centers, schools, hospitals, healers’ practices, private homes, etc.).

**Table 2:** Topic script

1. How is your health? What does it mean to you to be healthy? What does being healthy (or not) depend on? What do you do to be healthy?
2. Do you have any illnesses? If so, tell me about it. When you are sick, what do you do?
3. Do you usually go to a doctor or a traditional doctor? When? Where? Can you tell me about your experience?
4. Have you heard of HIV? What do you know about HIV? What is HIV? What does it consist of? How does the disease arise? What does it depend on if a person has HIV? Do you know anyone with HIV?
5. How do you know if a person has HIV? Do you know what the treatment is? Is there a cure? Would you go to a doctor or a traditional practitioner?
6. If you have HIV, can you tell me about your experience please?

### Data analysis

Semi-structured individual interviews and focus groups were recorded in digital audio format. All recorded information was transcribed verbatim. A quality control analysis of the transcriptions was carried out, consisting of randomly selecting different paragraphs for verification. Grounded theory was applied as a method of analysis. Thus, the data analysis followed an inductive process: (1) reading and rereading the interview transcripts (open coding); (2) labeling the information according to categories and codes (open coding and axial coding); (3) performing a relational analysis of the categories and codes; and (4) describing the manifest and latent content of the categories and codes. The results were triangulated among different members of the research team, in line with Denzin’s [25] approach.

### Ethical considerations

The study adhered to the principles of the Helsinki Declaration and the Belmont Report. The protocol of the study was approved by the Ethical Committee for Research in the Humanities and Social Sciences of the Ministry of Health and Welfare of Equatorial Guinea. We also obtained the informed consent of each participant before undertaking the interviews and focal groups.

## Findings

A variety of perceptions and beliefs about HIV were identified among the interviewees. Not all participants had the same perceptions and beliefs regarding the virus. The main perceptions of HIV were that it was: (1) a hospital disease; (2) a new and unknown disease; (3) a disease of others; (4) a disease that doesn’t exist; (5) a disease of bad luck; (6) a disease resulting from sexual abuse; (7) a contagious disease; (8) a deadly disease; (9) a strange disease; and (10) a chronic disease. Below we describe the content and meaning of these different perceptions of HIV.

### “A virus like a hospital disease”

Independently of gender, age, education and habitat, all interviewees perceived HIV as a “disease” of modern medicine: a “hospital disease”. It should be noted that in Equatorial Guinean society there are two types of disease: Fang illnesses, pertaining to traditional medicine, and hospital diseases, corresponding to modern medicine. Thus, there exists a dual perception of illness [26]. Since HIV is perceived as a “hospital disease,” interviewees explained that it could only be diagnosed by doctor of modern medicine. Therefore, they said, neither witchcraft nor traditional healers could treat or cure it:

“I asked and they told me that AIDS is a hospital disease that Fang medicine can’t cure” (male, HIV-positive).

“No, no, no, the healers cure illnesses. No, no, no, the healers cure traditional illnesses like curses, traditional things too, but they can’t cure malaria, they can’t cure AIDS, they can’t cure other diseases, many diseases they can’t cure” (woman, without diagnosis).

### “A virus like a new and unknown disease”

Interviewees from the older age group perceived HIV as a “new” and “unknown” illness. They explained that this illness had not previously existed in the country and that people had only recently begun to talk about it. They also said that there was hardly any information on the subject, and therefore its origins were unknown. According to some participants with a high level of education, this was one of the reasons why the existence of HIV was called into question in the country:

“I’m not from the time of Obiang, I’m from the time of Ondó Edu, who was regional president, my eyes opened first in the time of Macias. At that time, we knew nothing about AIDS. AIDS is something new. So, we don’t know this illness” (woman, without diagnosis).

“It’s not clear to me either, but I’d say, as we’ve always said: no, in all the time we’ve lived here, these illnesses haven’t existed” (male, without diagnosis).

### “A virus like a disease of others”

Interviewees living in villages and using traditional medicine had the perception that HIV was a “disease of others”, based on the belief that the virus was a disease imported to Guinea Equatorial by visiting white people. They explained that the disease could also enter the country with Guineans who travelled to Europe and later returned with it:

“They say not… They’re Western things and bad things. People who’ve been there bring back these lies that they have there. When a person thinks that way, I don’t stay with them because they’re never going to understand me […]. Also, I don’t understand why they don’t want to believe it even though they can see it” (male, without diagnosis).

“There are many people who come to bring benefits to Guinea, engineers, people with PhDs and so on, it’s a good thing for Guinea, but there are others who bring diseases. That’s the bad thing we suffer from now […]” (male, without diagnosis).

### “A virus like a disease that doesn’t exist”

None of those interviewed denied the existence of HIV or AIDS. However, some commented that there was a part of the Guinean population –mainly those with a low level of education– who denied the existence of HIV and AIDS in the country:

“Let’s not forget how famous AIDS is getting, right? Only blind and stubborn and skeptical people want to deny that it exists, but men of good heart obviously realize that it really exists” (male, without diagnosis).

Interviewees with a higher level of education stated that since part of the population denied the existence of HIV, patients diagnosed as HIV-positive in hospitals often questioned the results of the diagnosis. They explained that in many cases these patients saw it as an economic issue, thinking that health workers diagnosed them with HIV to make money from the treatment:

“Well, because they say that if they have an appointment at the hospital because of the virus it’s to pay them money and I don’t know what. Well, I don’t know how it is they don’t believe, despite the negative effects of AIDS that we’re seeing. Many insist, as Don Rigoberto said, that they don’t want to see the existence of AIDS and say that the doctors told them that to make money” (male, without diagnosis).

This profile of participants explained that the reasons why this population denied HIV were: (1) the lack of knowledge and information the population had about the virus and AIDS, since it was a “new disease”; and (2) the belief that it was a “disease of others”.

### “A virus like a disease of bad luck”

Another perception of HIV emerging among participants was that it was a “disease of bad luck”. This view prevailed among participants living in villages and using traditional medicine, although it was also found among other interviewees.

Those perceiving HIV as a “disease of bad luck” stated that the virus was a disease that depended on fate. It depended on what bad fortune had in store for the person, since they explained that it was a disease that no one desired and that no one sought to have. It was a disease that fell on the person like a curse:

“It’s bad luck, I don’t know where it came to me from. I, truthfully, and it’s not confirmed that I have HIV and AIDS, you understand? In all sincerity, I don’t agree with these analyses” (male, HIV positive).

In the discourses of those who held this perception of HIV as a “disease of bad luck,” a series of reasons underlying this perception emerged:

1. One was the possibility, and therefore the probability, of having been infected (or not) through sexual relationships, since this may or may not have happened, given that there are other ways of transmission:

“Maybe yes and maybe no, because perhaps there was some carelessness when having sex. Well, it might not be through sexual relationships, because it can be through a manicure or other ways […] but when it comes to you, you must accept it” (male, HIV positive).

2. Another was the possibility, and therefore the probability, that a person could be infected with the virus because there were ways of transmission that did not depend on their personal responsibility, such as pedicurists or hairdressers’ knives:

“It’s a disease of bad luck, you can get infected with HIV and AIDS through sexual relationships, or because on a whim you go and have a manicure. You don’t know who has manicures there. If someone infected with HIV and AIDS has just had a manicure and then they give you one, you get HIV and AIDS, it’s bad luck” (woman, HIV positive).

3. Another reason stemmed from the view of chance the population has regarding Fang illnesses, in general derived from animism [27]:

“[…] I think because… OK, from time to time I ask why exactly someone looked at me so that I got this disease. That’s what I think sometimes” (woman, HIV positive).

### “A virus like a disease of sexual abuse”

Another extremely widespread perception among the population interviewed was that HIV was a disease that was a consequence of “the abuse of sex”. Thus, they explained that it was a sexually transmitted disease. Having HIV was identified with having led a sexually promiscuous life. Hence many of the women interviewed made moral judgments about AIDS throughout their interviews, stressing that it was “a disease that was not good”. This perception was more prevalent among women than men:

“Well, the opinion I can have about HIV/AIDS is that it’s a disease caused in the places where there’s abuse of sex, where there’s abuse of sex. It’s the way people live now, it’s disorder, disorder in their lives” (male, HIV positive).

### “A virus like a contagious disease”

Another majority perception was that HIV was a “contagious disease”. Almost all interviewees stated that the virus was transmitted through the sexual act. However, to a lesser extent, other ways of transmission were referred to, such as blood on hairdressers’ knives for cutting hair, or shaving, pedicures and manicures. Some stated that HIV could be transmitted by plates, glasses and cutlery used by HIV-positive people. This perception contributed to creating mistrust and fear around the virus:

“So, we know that it’s a very dangerous disease, and very contagious throughout the world, because it’s in the world” (male, without diagnosis).

“If she’s touching this food with her hand, I’m not going to go and share with her. And if someone comes to me with a glass, then we wash the glasses” (woman, HIV positive).

However, in the discussion groups the debate over whether it was men or women who infected more people with the virus arose spontaneously. On this issue participants in the groups were unanimous in the opinion that it was more often men who infected women. Paradoxically, though, most women agreed that society blamed them, whether as wives or girlfriends, when a man was diagnosed as HIV positive:

“Women, often, when they already have a husband, they don’t know other men after that. But the men are the ones who are in the street, if they get HIV and bring it home to you, you can’t know where they’ve brought it from. What usually happens is that when the husband has HIV, people say that it’s the woman who has committed adultery” (woman, without diagnosis).

However, most interviewees perceived that women were those who suffered more HIV and/or AIDS. They explained that this was due to the greater promiscuity of Guinean males and their refusal to use contraceptives with their wives:

“Yes, because if we look at the statistics, if we look at the statistics of AIDS contagion, there are more women because they’re above the men. If we think about the traditional aspect of the area I live in, 50 meters from here, there’s a famous medicine woman, who sometimes when I pass her house has 67 ill women and only two men in there. Think about it” (male, no diagnosis).

### “A virus like a deadly disease”

With the exception of participants with a higher level of education, another majority perception was based on the idea that HIV was fatal. Interviewees explained that it was a very dangerous disease, which had no cure and inevitably led to death:

“Yes, I knew that it was a dangerous disease, a bad one. In any case, well, I knew that this illness has no future. Well, I thought, when I saw I was infected I wouldn’t live any longer. That’s why I wanted to die, before…” (woman, HIV positive).

Participants with a higher level of education, and some other interviewees, commented that the Guinean population believed that when someone was infected with HIV, “death comes to them suddenly”:

“It will be right now, a sudden death shall we say” (male, without diagnosis).

“It’s not the same, how can it be the same, death comes to you” (male, without diagnosis).

Some of the women interviewed, however, questioned this belief in sudden death. They stated that they knew people with AIDS who had been living for some time and who were even working and bringing home money.

Interviewees with a higher level of education commented that the association between HIV and death had caused a lot of social alarm and hysteria around HIV in Guinean society. This meant that the “disease” had not been normalized in Guinea as it had in European countries:

“What has most provoked disgust about this disease in our society is that it’s been dramatized a lot. I’m sure that that a Western person, an American doesn’t have the same concept of AIDS as an African. It seems that they’ve told Africans that AIDS is a disease that whoever carries it is apt to die at any time” (male, without diagnosis).

The fact that the virus is perceived as a “deadly disease” stems from the identification of HIV with AIDS. In this respect it should be noted that most interviewees did not distinguish between the two terms. They were not aware of any clinical distinction between acquiring the virus and suffering from acquired immune deficiency syndrome. They equated being an HIV-positive person with having AIDS; hence the idea that HIV was fatal. Therefore, due to the identification of HIV with AIDS, none of the HIV-positive people interviewed responded in the affirmative when asked if they suffered from HIV:

Interviewer: “What is HIV?”

C: “They say that it’s AIDS, right? Isn’t it AIDS?”

Interviewer: “Timothy, what do you think?”

T: “I have that idea because I don’t know what AIDS means, if it’s malaria I don’t know what it means. I have that idea, a disease called AIDS, I don’t know.”

Interviewer: “Nicholas?”

N: “The same for me, I don’t know what the disease called AIDS is, I only hear AIDS, AIDS” (men, without diagnosis).

### “A virus like a strange disease”

Another perception of HIV held by some interviewees was that it was a “strange disease.” Interviewees with a higher level of education said that since HIV was seen as a “hospital disease”, but one which did not disappear from the body when pharmaceutical medicines were taken, or since it had no cure (as opposed to other “hospital diseases” like malaria), then the idea of the disease as an anomaly was accentuated:

“Because you can’t cure it. On that basis it can’t be the same as malaria because it seems that you can cure malaria […]” (male, HIV positive).

“The illness that most worries me because this illness that we’ve got now that’s called AIDS doesn’t have a cure. I can get ill with any other illness, all other diseases have a cure” (woman, without diagnosis).

### “A virus like a chronic disease”

Lastly, participants with a higher level of education perceived HIV as a “chronic disease”. They stated that thanks to medical advances, specifically the development of antiretrovirals, the virus was now not a “deadly disease” but a chronic one:

“With so many advances they were saying that they were taking antiretrovirals, which means that now it’s not fatal. They cost 8,000 francs a month, well, that’s information from the TV” (male, without diagnosis).

They also commented that thanks to the improvements and successes achieved in the country, HIV was now a “chronic disease”. They referred particularly to the greater accessibility of treatment, made possible by the government’s free distribution of antiretrovirals, as an important factor.

Also, interviewees with a higher level of education and some others commented that the different perceptions of HIV among the population depended on their level of education: the higher the level of education, the less it was perceived as a “deadly disease” and the more it was seen as normal and the diagnosis accepted:

“The less educated population still sees it as a deadly disease, although it’s also deadly, but those who are better informed see it as a chronic disease if you’re in treatment” (male, without diagnosis).

## Discussion

The findings in this study show the coexistence of different perceptions and meanings of HIV amongst the population of Equatorial Guinea. In this respect, as with other studies undertaken in the African context [28], our research demonstrates that age, place of residence and level of education all influence the way HIV is perceived. Nevertheless, it is important to mention that some meanings ascribed to HIV were shared by the majority of the population. These findings are useful for addressing the need to boost the impact of primary prevention amongst the population; and it is therefore recommendable that the contents of HIV information campaigns should take into account the heterogeneity of meanings and perceptions existing amongst the different population groups.

The existence of different perceptions of HIV demonstrates the strong influence that cultural context, especially Fang culture, has on the construction of meanings around the virus. Numerous studies have corroborated the influence of the cultural context on the perception of HIV in African countries [29–31]. In this sense, the influence of the local cultural would explain why the virus is perceived by older people as a “new and unknown disease”, since this minority perception is based on the fact that HIV does not correspond to any of the diseases of Fang culture. The perception of HIV as a “disease of others” also acquires meaning in terms of the local cultural setting.

This perception can have an impact on risk perception. As HIV is seen as a “disease of others”, people may minimize the likelihood of infection and therefore not take preventive measures. In the light of these findings, then, it is recommended to undertake, among this sector of the population, campaigns of education and information based on peer methodology, i.e., using HIV-positive local peers with the purpose of demystifying the false belief that HIV is a “disease of others”. The fact that the virus is perceived as a non-traditional disease –and therefore a new, unknown illness that belongs to others—has led part of the population to deny its existence. In this study, such denial seemed to correspond to the sector of the population which was older and had a lower level of education. Denial of HIV also clearly represents a major obstacle to the adoption of prevention practices, and therefore to the efficacy of prevention programs. Thus it is additionally recommended to undertake peer education initiatives among this population sector to address the false belief that HIV does not exist. Several such initiatives have already been carried out successfully in the African context [32, 33], for example peer-to-peer intervention in Ethiopia in sex education for HIV prevention [34].

A final minority perception of HIV as an “unlucky disease” is derived from the animist doctrine of Fang culture about the role of chance in the genesis of disease. This perception may also influence the perception of HIV risk. Some studies carried out in Africa have shown that the population living in rural areas has a perception of the virus that is much more distant from constructs of health and illness found in Western medicine [35–38]. Thus, a further recommendation would be to devise strategies to change this perception of risk.

The social representation [39] of HIV is constructed around the majority perceptions of the virus as a “hospital disease, a “deadly disease” and a “disease of sexual abuse”. The predominant perception of HIV as a “deadly disease” is a result of the association of HIV with AIDS, and this in turn is a consequence of the prevention campaigns carried out in the country. These campaigns replicated European models from the late 1980s and 90s, when AIDS had not yet become a chronic disease as no effective treatment had yet been developed [40, 41]. Campaigns undertaken in the mid-1990s in Equatorial Guinea reproduced this association between HIV, AIDS and fatality. Lastly, the majority perception of a “disease caused by the abuse of sex” is constructed on the basis that HIV is a sexually transmitted disease of modern medicine and is thus a result of moving away from Christian sexual norms and moral precepts. In this respect the scientific literature in the field shows that this normative aspect, apart from being moralistic with regard to sexuality, has also acted as a breeding ground for stigmatizing processes in other societies, for example in the West [42]. For example, this has been found in the case of European and American homosexuals, who have been strongly stigmatized by the association of HIV with leading a promiscuous sexual lifestyle [43].

Majority perceptions have given rise to the prevailing social representation of HIV in Equatorial Guinean society. This representation is based fundamentally on the identification of HIV with the condition of AIDS and on the perception of it as “deadly disease” and as “a disease caused by the abuse of sex”. This social representation can present barriers to the acquisition of preventive practices such as voluntarily undergoing early diagnosis. When HIV is associated with death, the results of diagnostic tests acquire an overwhelming importance. Therefore, a further recommendation stemming from these findings is to implement strategies to dismantle this social representation and to take it into account when designing prevention programs.

Although information and knowledge about HIV are a necessary precondition for the adoption of preventive practices, they are now consistently shown to be insufficient if many other social and cultural factors are not taken into account. HIV prevention strategies, then, need to embrace the conceptual model of the social determinants of health [44]. In this area, our findings indicate that the factors most influencing the prevalence of the epidemic in Equatorial Guinea are the structural determinants of health inequalities, as in many African countries [45, 46]. Among such structural factors, the political and socio-economic context in which public policies are developed, together with the culture and values of the society itself, occupy the first place. For example, the insufficiency of policies to promote health, or to control health levels, together with the low value placed on health amongst the Guinean population, mean that the population is generally in a poor state of health*.* Also, the lack of value that health has in society is accentuated by the mystical and spiritual conception of illness, of whatever type, which exempts the individual from responsibility for protection and care in their own health and illness, including HIV; and this in turn leads to the disempowerment of health. A similar mystical conception of illness is also held in other African countries, such as South Africa [47, 48]. In this regard, and in relation to HIV, it is clear that axes of inequality such as gender, ethnicity and educational background are determining factors in Guinean society*.* These factors are also highly explanatory in the distribution of HIV prevalence in many sub-Saharan African countries such as Angola, Mozambique, etc., as shown by the results of a systematic literature review [49, 50]. However, in terms of sex, although it has been scientifically proven that there is a higher biological risk for women rather than men during unprotected vaginal intercourse [51], higher prevalence in women is more gendered in relation to social stratification and the distribution of power, as these factors place them in a situation of greater social vulnerability to HIV. As a result of high prevalence rates among women in Africa, there is talk of the “feminization of HIV” [52]. In the specific case of Equatorial Guinea, dowry is a strong barrier to the acquisition of male condom use for HIV prevention. Regarding ethnicity, and specifically tribal identity, which determines economic status as an axis of inequality, it is worth noting that both high and low economic status increase the risk of becoming infected with the virus.

### Limitations of the study

The findings of this study represent only the Fang ethnic group. Hence, they cannot be extrapolated to the other ethnic groups living in Equatorial Guinea. Neither did our sample include youth (ages 15–25), women with a higher level of education, or the population with a high economic level.

### Future lines of research

One interesting line of future research would be to replicate the study in the island area and in the interior of the country. Other population groups that would be highly relevant to research, since no studies have been conducted among them to date, are homosexuals, female sex workers and the military.

## Conclusions

The local cultural context exercises a strong influence on the construction of the different meanings ascribed to HIV in Equatorial Guinean society. The existence of different perceptions of the virus, and its social representation, can constitute major barriers to the efficacy and effectiveness of prevention strategies. Relationships exist between demographic variables and the specific meanings given to the virus. Therefore, for greater effectiveness, this heterogeneity and these relationships should be considered in strategic planning. Information strategies should also be designed taking into account the social determinants of health that exist amongst the population.

### Supplementary Information

**Additional file 1. **

## Data Availability

The data generated and/or analyzed during the present study are not publicly available, due to our wish to maintain the confidentiality of the HIV diagnosis among the patients participating, since it is a disease with strong stigma. However, the data are available from the corresponding author on reasonable request.
