# Supplementary material for: “It doesn’t exist, only other people have it, or it’s bad luck”: perceptions of HIV as barriers to its prevention in Bata
Source: BMC Public Health. 2023 Nov 27;23:2347. doi: 10.1186/s12889-023-17215-0 (PMC10683102; doi:10.1186/s12889-023-17215-0)
Supplement: Supplementary file 1 — Additional file 1. [file 12889_2023_17215_MOESM1_ESM.docx]

**“Virus like a hospital disease”**

“I asked and they told me that AIDS is a hospital disease that Fang medicine can’t cure” (Male, HIV-positive).

“No, no, no, the healers cure illnesses. No, no, no, the healers cure traditional illnesses like curses, traditional things too, but they can’t cure malaria, they can’t cure AIDS, they can’t cure other diseases, many diseases they can’t cure” (Women, without diagnosis).

**“Virus like a new and unknown disease”**

“I’m not from the time of Obiang, I’m from the time of Ondó Edu, who was regional president, my eyes opened first in the time of Macias. At that time, we knew nothing about AIDS. AIDS is something new. So, we don’t know this illness” (Woman, without diagnosis).

“It’s not clear to me either, but I’d say, as we’ve always said: No… in all the time we’ve lived here, these illnesses haven’t existed” (Male, without diagnosis).

**“Virus like a disease of others”**

“They say not… they’re Western things and bad things. People who’ve been there bring back these lies they have there. When a person thinks that way, I don’t stay with them because they’re never going to understand me […]. Also, I don’t understand why they don’t want to believe it even though they can see it” (Male, without diagnosis).

“There are many people who come to bring benefits to Guinea, engineers, people with PhDs and so on, it’s a good thing for Guinea, but there are others who bring diseases. That’s the bad thing we suffer from now […]” (Male, without diagnosis).

**“Virus like a disease that doesn’t exist”**

“Let’s not forget how famous AIDS is getting, right? Only blind and stubborn and skeptical people want to deny that it exists, but men of good heart obviously realize that it really exists” (Male, without diagnosis).

“Well, because they say that if they have an appointment at the hospital because of the virus it’s to pay them money and I don’t know what…Well, I don’t know how it is they don’t believe, despite the negative effects of AIDS that we’re seeing… Many insist, as Don Rigoberto said, that they don’t want to see the existence of AIDS and say that the doctors told them that to make money” (Male, without diagnosis).

**“Virus like a disease of bad luck”**

“It’s bad luck, I don’t know where it came to me from. I, truthfully, and it’s not confirmed that I have HIV and AIDS, you understand? In all sincerity, I don’t agree with these analyses” (Male, HIV positive).

“Maybe yes and maybe no, because perhaps there was some carelessness when having sex. Well, it might not be through sexual relationships, because it can be through a manicure or other ways […] but when it comes to you, you must accept it” (Male, HIV positive).

“It’s a disease of bad luck, you can get infected with HIV and AIDS through sexual relationships, or because on a whim you go and have a manicure. You don’t know who has manicures there. If someone infected with HIV and AIDS has just had a manicure and then they give you one, you get HIV and AIDS, it’s bad luck” (Woman, HIV positive).

“[…] I think because… OK, from time to time I ask why exactly someone looked at me so that I got this disease… that’s what I think sometimes” (Woman, HIV positive).

**“Virus like sexual abuse disease”**

“Well, the opinion I can have about HIV/AIDS is that it’s a disease… caused in the places where there’s abuse of sex, where there’s abuse of sex. It’s the way people live now, it’s disorder, disorder in their lives” (Male, HIV positive).

**“Virus like a contagious disease”**

“So, we know that it’s a very dangerous disease, and very contagious throughout the world, because it’s in the world” (Male, without diagnosis).

“If she’s touching this food with her hand, I’m not going to go and share with her. And if someone comes to me with a glass, then we wash the glasses” (Woman, HIV positive).

“Women, often, when they already have a husband, they don’t know other men after that. But the men are the ones who are in the street, if they get HIV and bring it home to you, you can’t know where they’ve brought it from. What usually happens is that when the husband has HIV, people say that it’s the woman who has committed adultery” (Woman, without diagnosis).

“Yes, because if we look at the statistics, if we look at the statistics of AIDS contagion, there are more women because they’re above the men. If we think about the traditional aspect of the area I live in, 50 meters from here, there’s a famous medicine woman, who sometimes when I pass her house has 67 ill women and only two men in there. Think about it” (Male, no diagnosis).

**“Virus like a deadly disease”**

“Yes, I knew that it was a dangerous disease, a bad one. In any case, well, I knew that this illness has no future. Well, I thought, when I saw I was infected I wouldn’t live any longer. That’s why I wanted to die, before…” (Woman, HIV positive).

“It will be right now, a sudden death shall we say” (Male, without diagnosis).

“It’s not the same, how can it be the same, death comes to you” (Male, without diagnosis).

.

“What has most provoked disgust about this disease in our society is that it’s been dramatized a lot. I’m sure that that a Western person, an American doesn’t have the same concept of AIDS as an African. It seems that they’ve told Africans that AIDS is a disease that whoever carries it is apt to die at any time” (Male, without diagnosis).

:

Interviewer: “What is HIV?”

C: “They say that it’s AIDS, right? Isn’t it AIDS?”

Interviewer: “Timothy, what do you think?”

T: “I have that idea because I don’t know what AIDS means, if it’s malaria I don’t know what it means. I have that idea, a disease called AIDS, I don’t know.”

Interviewer: “Nicholas?”

N: “The same for me, I don’t know what the disease called AIDS is, I only hear AIDS, AIDS” (Men, without diagnosis).

**“Virus like a strange disease”**

“Because you can’t cure it. On that basis it can’t be the same as malaria because it seems that you can cure malaria […]” (Male, HIV positive).

“The illness that most worries me because this illness that we’ve got now that’s called AIDS doesn’t have a cure. I can get ill with any other illness, all other diseases have a cure” (Woman, without diagnosis).

**“Virus like a chronic disease”**

“With so many advances they were saying that they were taking antiretrovirals, which means that now it’s not fatal. They cost 8,000 francs a month, well, that’s information from the TV” (Male, without diagnosis).

“The less educated population still sees it as a deadly disease, although it’s also deadly, but those who are better informed see it as a chronic disease if you’re in treatment” (Male, without diagnosis).
